# Unraveling the Roles of Regulatory Genes during Domestication of Cultivated *Camellia*: Evidence and Insights from Comparative and Evolutionary Genomics

**DOI:** 10.3390/genes9100488

**Published:** 2018-10-10

**Authors:** Chao Yan, Ping Lin, Tao Lyu, Zhikang Hu, Zhengqi Fan, Xinlei Li, Xiaohua Yao, Jiyuan Li, Hengfu Yin

**Affiliations:** 1 State Key Laboratory of Tree Genetics and Breeding, Research Institute of Subtropical Forestry, Chinese Academy of Forestry, Hangzhou 311400, Zhejiang, China; yanc01@163.com (C.Y.); linping80@126.com (P.L.); taolyu1@163.com (T.L.); huzhikang01@163.com (Z.H.); fanzhengqi_risf@outlook.com (Z.F.); lixinlei_risf@outlook.com (X.L.); yaoxh168@163.com (X.Y.); lijiyuan_risf@outlook.com (J.L.); 2 Key Laboratory of Forest Genetics and Breeding, Research Institute of Subtropical Forestry, Chinese Academy of Forestry, Hangzhou 311400, Zhejiang, China; 3 Experimental Center for Subtropical Forestry, Chinese Academy of Forestry, Fenyi 336600, Jiangxi, China; 4 College of Marine Sciences, Ningbo University, Ningbo 315211, Zhejiang, China

**Keywords:** domestication, genomics, *Camellia*, transcription factors, quantitative trait locus, genome-wide association study, fruit development

## Abstract

With the increasing power of DNA sequencing, the genomics-based approach is becoming a promising resolution to dissect the molecular mechanism of domestication of complex traits in trees. Genus *Camellia* possesses rich resources with a substantial value for producing beverage, ornaments, edible oil and more. Currently, a vast number of genetic and genomic research studies in *Camellia* plants have emerged and provided an unprecedented opportunity to expedite the molecular breeding program. In this paper, we summarize the recent advances of gene expression and genomic resources in *Camellia* species and focus on identifying genes related to key economic traits such as flower and fruit development and stress tolerances. We investigate the genetic alterations and genomic impacts under different selection programs in closely related species. We discuss future directions of integrating large-scale population and quantitative genetics and multiple omics to identify key candidates to accelerate the breeding process. We propose that future work of exploiting the genomic data can provide insights related to the targets of domestication during breeding and the evolution of natural trait adaptations in genus *Camellia*.

## 1. Introduction

Domestication of wild plants has resulted in substantial phenotypic changes related to molecular changes of key regulatory genes. Understanding the genetic basis of domestication can provide insights into the mechanism of rapid evolution of traits influenced by human demands. Using forward genetics, researchers have identified many important genes contributing to various processes relevant to plant development and growth in several crop species. In addition, to achieve some human desirable traits, mutations of homologous genes in different plants have occurred [1,2]. For instance, the domestication of the loss of seed shattering in sorghum is found to be controlled by mutations of a YABBY transcription factor [3] and the modifications of orthologs in rice and maize were revealed to be involved in shattering domestication [3]. Therefore, convergent selection of gene alleles related to important regulators provides a promising insight into revealing the trait domestication in different selection programs of plants [4,5].

However, in perennial woody plants, the approach from phenotypes to genetic modification is merely successful due to the long breeding cycles and inbreeding depression. Recent advances in genomics have facilitated the unraveling of the evolution history and selection signatures of cultivated tree crops. In fruit trees, genome sequencing, and re-sequencing have provided essential evidence of genomic signatures of domestication such as gene family expansion, genomic diversity, genome selective sweep and more [6,7,8,9]. In a recent study, combined analyses of large-scale re-sequencing and transcriptomics in pears have led to the identification of valuable candidate genes underlying fruit quality [10]. Therefore, the genome sequencing and global expression analyses in tree plants provide a useful means for understanding how traits are domesticated and how they have evolved. 

The genus *Camellia* includes more than 250 species and is the largest genus in the plant family Theaceae [11,12]. *Camellia* species are generally evergreen shrubs or small trees and most of them originate from Southeastern Asia. A majority of these species is native to China. Despite the disagreements of different taxonomic systems, cultivars of wild *Camellia* species are currently known all over the world. In *Camellia* plants, the human selection of favorable traits such as leaf metabolism, floral development, and seed oil content has resulted in substantial genetic alterations in commonly-seen cultivars when compared to their wild ancestors. The understanding of the genetic and genomic characteristics under the breeding process is of great significance for dissecting trait evolution and domestication to facilitate *Camellia* breeding.

**Figure 1:**
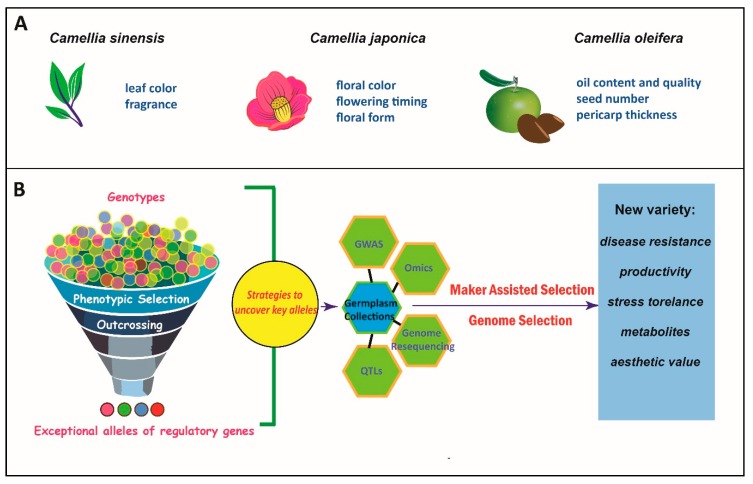
An illustrated cartoon summarizing the domestication in *Camellia* species. (**A**) The leaves, flowers, and fruits (seeds) of *Camellia* plants are useful organs to produce economic products for human living. Major domestication targets of each organ and representative species are listed. (**B**) The domestication process yields some valuable alleles contributing to trait variations in cultivars. To identify underlying genes or associated molecular makers, strategies based on germplasm collection (natural or forced hybridization populations) are subjected to various types of analyses such as genome-wide association study (GWAS), multiple omics tools, genome re-sequencing, and quantitative trait locus (QTL) mapping. The uncovered markers and genes associated with key traits are pivotal for understanding the mechanism of domestication and improving new varieties in *Camellia*. With the support of maker assisted selection and genome selection approaches, the domestication program of *Camellia* plants can be more efficiently and precise for breeding cultivars of economic values.

In recent years, the breakthrough of DNA sequencing technology has greatly expedited the basic research of understanding the molecular domestication process in *Camellia*s. Progress in generating high-quality and in-depth genomics databases were made in *Camellia* plants. Particularly, two reference genomes of tea trees were released [13,14], which could facilitate the future research in unraveling the mechanisms of evolution and domestication of *Camellia* plants. A number of RNA-sequencing (RNA-seq) studies were reported in several wild and cultivated *Camellia* plants. In this review, we aim to provide a timely summarization of advances in genomic and genetic studies of large-scale gene expression profiling (Table 1). We go over recent progresses of genomics research in *Camellia* plants and focus on pathways related to traits with significant economic values. We survey the studies utilizing high-throughput sequencing technology for investigating global gene expression profiling, small RNA identification, and other types of protein coding and noncoding genes. We also pay a close look at the field of population genetics approaches in *Camellia* plants including the genome-wide association study (GWAS) and quantitative trait locus (QTL) mapping analyses even though research at present still lacks a meaningful scale to isolate key DNA regions (Figure 1). We propose that building up high-quality reference genomes in several representative species is pivotal for molecular dissection of domestication and for promoting the breeding of new varieties. Furthermore, the system biology approach integrated diverse analyses of high-throughput omics datasets, which will accelerate the discovery of key regulatory genes.

## 2. Genetics and Genomic Resources in Genus *Camellia* Empowered by High-Throughput Sequencing 

Genus *Camellia* displays an extraordinary natural diversity in morphology, metabolites, habitats, etc. The artificial breeding of *Camellia* species results in excellent cultivars for producing beverages, ornamental flowers, and edible oil (Figure 1). These are the main purposes of breeding and cultivation of *Camellia* varieties and the related research is also carried out around these breeding targets. *Camellia sinensis* var. *assamica* was the first species in *Camellia* with a high-quality genome reference in which 3.02-Gb base pairs DNA sequences were assembled [14]. In 2018, the genomic sequence of *C. sinensis* var. *sinensis* was reported [13]. Through the study of two *C. sinensis* genomes, the researchers found that whole-genome duplication events and subsequent paralogous duplications had major impacts on the gene family members related to the biosynthesis of secondary metabolites such as catechins, theanine, and caffeine [13,14]. The high-quality genomes in *Camellia* will greatly facilitate fundamental research relevant to trait variations through the analyses of comparative and functional genomics. 

Transcriptomics studies in various *Camellia* plants were performed extensively in both wild and cultivated plants. The first comprehensive transcriptome analysis in *C. sinensis* by the *second generation sequencing* was reported in 2011 using an Illumina GA II sequencing platform [15]. This work sequenced a mixed sample containing seven tissue types and yielded 127,094 uni-genes, which provided a dataset for gene discovery not only for tea plants but also other *Camellia* species [15]. Along with the rapid development of sequencing technology, the RNA-seq studies of *Camellia* were applied dramatically to enhance our understanding of how genes are expressed under various conditions. In this case, we focus on transcriptomics studies in *Camellia* plants under a variety of experimental designs (Table 1). 

### 2.1. Responses of Biotic and Abiotic Stress in Camellia Plants

Various RNA-seq analyses targeting biotic and abiotic stresses in *C. sinensis* were performed to identify responsive genes. In those RNA-seq analyses, the majority of them characterize abiotic stress responsive genes including temperature, drought, salinity, nitrogen, etc. Genes responsive to a low temperature were identified in *C. sinensis* by several RNA-seq analyses, which revealed some potential signaling pathways underlying cold acclimation [16]. Among the 1770 differentially expressed transcripts, potential cold sensors, detoxification enzyme genes, and transcription factors were identified [16]. Based on the previously mentioned transcription database, the 89 APETALA2/ethylene responsive factor (AP2/ERF) and 50 putative WRKY proteins, which might be related to abnormal temperature stress, were identified [17,18]. In *Camellia japonica*, the cold responses were compared between cold sensitive and cold hardy genotypes through a transcriptomics analysis and α-linolenic acid and jasmonic acid biosynthesis pathways were proposed as key factors related to low temperature acclimation [19]. Salinity and drought tolerance is another focus of plant breeding and cultivation and has an important influence on plant growth [54]. To understand the gene expression profiles, the RNA-seq experiments under drought and salt stress treatments of *C. sinensis* were conducted, and 3936 and 3715 differentially expressed genes (DEGs) were identified upon drought and salt stress, respectively [20]. Further comparative analyses of DEGs indicated that these two stresses had some common molecular effects, which indicates that shared pathways were involved [20]. The seed germination process of *Camellia* was also found to be sensitive to drought stress. Through transcriptomics analysis under the dehydration treatments, 91,925 non-redundant uni-genes were obtained. A series of genes that have been reported to function in the dehydration process were found to be downregulated including ABA biosynthesis and signal transduction, transcription factor, antioxidant enzyme, etc. [21].

Nitrogen (N) is one of three essential factors in higher plants, which is critical for plant growth. The transcriptomes of buds, leaves, and the root of *C. sinensis* with or without (NH_4_)_2_SO_4_ treatments were studied and 196 and 29 common DEGs in roots and leaves were identified, respectively [22]. Through quantitative reverse transcription PCR (RT-qPCR) analysis, some N uptake and assimilation genes were validated [22]. Nitric oxide (NO) was found to be an important signaling factor regulating plant growth under a stress condition [55] especially in low temperature stress [56,57]. In order to reveal potential mechanisms of NO responses to a low temperature, transcriptomes of pollen tubes at 25 °C (CK) and 4 °C (LT) or with NO treatment (NO) were studied [23] and 766,497 and 929 DEGs were found among CK-VS-LT, CK-VS-NO, and LT-VS-NO comparisons [23]. These results provided molecular evidence of linking N assimilation and cold stress in *Camellia* plants. 

Most of *Camellia* species originate from Southeastern Asia, which has a warm and humid climate that is particularly suitable for pest and disease propagation. In such an environment, *Camellia* plants generally have strong biotic resistance. The disease resistance ability is an important index in the *Camellia* plant Breeding. Artificial cultivation of a single species weakened *Camellia* plant biotic resistance. Some diseases and insect pests seriously affect the economic value of *Camellia* plants such as tea blister blight (BB), anthracnose, *Ectropis oblique*, etc. The tea BB has a great influence on the quality of tea [58]. The molecular mechanism of the *C. sinensis* defense against the BB disease was studied [24]. The transcriptomes of BB transition leave tissues were analyzed along with 149 DEGs including defense related enzymes, resistance genes, multidrug resistant transporters, etc. It was suggested that they have a role in defending against BB [24]. Anthracnose is one of the most important diseases restricting the development of Oil *Camellia* species [59]. The correlations of *Camellia oleifera* disease resistance and the enzyme (peroxidase, catalase, superoxide dismutase, and polyphenol oxidase) activity were analyzed. It is found that infection of pathogenic bacteria caused the significant changes of the activity of defensive enzymes in different *Camellia* varieties [60]. To elucidate the molecular mechanisms of the response to *E. oblique* in tea plants, the transcriptomes of *E. oblique* damage-induced leave tissues were analyzed and 1859 DEGs were identified [25]. Upon further analysis, this DEGs found that genes involved in secondary metabolites and signaling pathways were differentially regulated after feeding by *E. oblique* [25].

### 2.2. Transcriptomic Analyses in the Control of Secondary Metabolism in Camellia

Transcriptomics studies were applied to understand various developmental processes of *Camellia* plants. Particularly in tea plants, global gene expression profiling along with the development of leaf, flower, and seed germination were effective in identifying potential regulatory genes (Table 1). *Camellia* is rich in many secondary metabolites including flavonoids, catechins, theanine, anthocyanins, etc. Many of these secondary metabolites are not only essential to the flavors of tea drinks but also have a significant impact on horticultural value of *Camellia*. There were many RNA-seq studies related to secondary metabolites of *Camellia*. For example, the transcriptomes of 13 different samples from various organs and developmental stages of *C. sinensis* were studied and 347,827 uni-genes were assembled and annotated in which 1719 uni-genes were identified as being involved in the secondary metabolic pathways [26]. In *Camellia taliensis*, the transcriptomes of tender shoots, young leaves, flower buds, and flowers were studied and candidate genes for major metabolic pathways were found [27]. The biosynthetic pathway of catechins were also studied [28,29]. Through the transcriptomics analysis of leaf tissues from four tea plant cultivars (*C. sinensis*), 146,342 pairs of putative orthologs were generated and 217 common DEGs were found [29]. A similar work characterized the four tissue types of *C. sinensis* in which 36 catechins and flavonoids biosynthesis were identified [28]. These studies indicated that the biosynthesis pathways of secondary metabolites in different *Camellia* species were largely conserved and the changes of regulatory genes might play a key role in the diversity of chemical compositions. 

The diverse pigments of *Camellia* plants are attractive to breeders. Some ornamental traits such as purple leaves, golden flowers, and double flowers were characterized through different transcriptomics approaches. To understand the alterations of the leaf color of *C. sinensis var. assamica*, a transcriptomics comparison between green and purple leaves was performed, which yielded 2250 DEGs that were potentially related to the biosynthesis of anthocyanins [30]. In yellow *Camellia* (*Camellia nitidissima*) species, the transcriptomes of floral buds at five different developmental stages were investigated and, through characterization of DEGs, the accumulation of carotenoids and flavonols glucosides in the petals was revealed as potential underlying floral pigments [31]. A recent work investigated two transcriptomic datasets in *C. nitidissima* and *Camellia chuongtsoensis* [32]. Comparative analyses of gene expression profiles in yellow and red flowered *Camellia* species revealed that the direct glucosidation of flavonols was a key regulatory step of accumulating yellow pigments [32].

### 2.3. Transcriptomics Studies Related to Floral Patterning, Flowering Timing, and Bud Dormancy

The double flower formation in *Camellia* is a major aesthetic trait for ornamental *Camellia* plants. An integrative analysis using small RNA, transcriptome, and degradome sequencing technologies in wild and domesticated double flower cultivars was performed [33]. Through gene expression and functional enrichment analyses, the formation of double flowers was found to be controlled by a coordination of micro RNAs (miRNAs) and some floral regulators [33]. There were only a few works reported in *Camellia* characterizing the miRNA genes. Through small RNA-seq of different floral organs in *C. azalea*, 175 miRNAs were identified and 12 *Camellia* specific miRNAs were revealed [34]. The differentially expressed miRNAs and their targets were analyzed and some miRNA-target regulations were identified as important factors in the control of floral organ development in *Camellia* [34].

Bud dormancy is an important evolutionary adaptation to local climatic conditions, which enables the survival in winter or dry seasons. In *C. sinensis*, cold winter dormancy also affected the economic output of the tea plant [35]. The transcriptomes of bud tissues (*C. sinensis*) of different developmental stages were studied. The putative role of identified genes in growth and dormancy of tea were discussed [36]. Transcriptomes of axillary buds (*C. sinensis*) of different dormancy types and bud flush stages were identified and 16,125 DEGs were identified in different measured conditions, which suggested that the dormancy regulation of the tea plant were consistent with that of Poplar [35]. In *Camellia azalea*, which is a newly discovered species with unique and prolonged blooming periods, the transcriptomes of floral buds at different developmental stages were investigated and the dormancy associated MADS-box genes were further analyzed, which showed that *Short Vegetative Phase* (*SVP*-) and *Agamous-like24* (*AGL24*-) genes played critical roles during floral bud development [37]. In *Camellia sinensis*, a transcriptomic analysis of flower development revealed several types of transcription factors including *WRKY*, the *Ethylene Responsive Factor* (*ERF*), the *basic Helix-Loop-Helix* (*bHLH*), the *Myeloblastosis* (*MYB*), and the *MADS-box* family, which were related to floral transition [38].

Some other transcriptomic studies were conducted to understand environmental or hormonal regulations in *Camellia* plants. In order to understand the non-deciduous habit of *C. sinensis*, transcriptomes of buds in July and December were analyzed and 24,700 uni-genes were obtained, which were related to the operation of winter tolerance [39]. In a transcriptomics study of shading treatment of “Baijiguan” (*C. sinensis*) leaves, 1993 and 2576 DEGs were identified in plants treated with three and six days of shading, respectively [40]. It is postulated that the high light intensity might affect PSII stability, chloroplast development, and chlorophyll biosynthesis by inhibiting the expression of the *photosystem II10-k Da protein* (*Psb R*) [40]. The transcriptomes of adventitious roots (*C. sinensis*) with and without Indole-3-Butyric Acid treatment were compared [41,42] and 656 up-regulated and 435 down-regulated genes were identified. Functional annotation analysis revealed the potential mechanism relevant to the control of the adventitious rooting process [42].

### 2.4. Transcriptomics in Oil Camellia Plants

Almost all of *Camellia* seeds have some certain oil contents. Oil *Camellia* referred to a kind of *Camellia* species that the main cultivated purpose was seeds oil content including *C. oleifera*, *Camellia meiocarpa*, *Camellia chekiangoleosa*, etc. *C. oleifera* was the most important species in oil *Camellia*. In *C. oleifera*, about 60 million RNA-Sequence reads from four tissues were generated and assembled into 104,842 non-redundant putative transcripts [43]. This work greatly increased the transcripts sequences for gene discovery and identified 3022 pairs of orthologs compared with *C. sinensis* [43]. The transcriptomes of *C. oleifera* leaves under drought treatments were studied. A series of DEGs associated with drought stress responsive pathways were identified among which 789 DEGs were transcription factors [44]. In a recent study, transcriptomes of *C. oleifera* seeds at different oil accumulation stages were characterized and valuable DEGs that were associated with the seed oil accumulation were uncovered [45]. By studying the transcriptomes of *C. oleifera* leaves at different elevations of Lu Mountain and Jinggang Mountain in China, abundant simple sequence repeats (SSRs), Single nucleotide polymorphisms (SNPs), and insertion/deletions were identified and many DEGs at different environmental temperatures were discovered [46].

Different varieties of oil *Camellia* had their own characteristics. In order to reveal the mechanism of phenotypic differences, the transcriptomes of various oil *Camellia* cultivars were analyzed. *C. chekiangoleosa* was a kind of oil *Camellia* with red flowers. In *C. chekiangoleosa*, RNA-Seq datasets and Expressed Sequence Tag (EST) library were generated to study genes involved in anthocyanin and seed oil biosynthesis [47]. The transcriptomes of *C. meiocarpa* and *C. oleifera* seeds, which have different moisture contents, were analyzed. In addition, 244 genes involved in fatty acid synthesis and accumulation were identified and gene ontology enrichment indicated that fatty acid accumulation is essential in *C. meiocarpa* and *C. oleifera* during the natural drying process [48]. A comparative study using *C. oleifera*, *C. chekiangoleosa*, and *Camellia brevistyla* was performed. The expression levels of *Chalcone Synthase* (*CHS*) and *Fatty Acid Desaturase 2* (*FAD2*) were compared in different cultivars [49].

### 2.5. Markers Development Based on RNA-Sequencing

RNA-sequence data were widely used in the development of the molecular markers of *Camellia*. To facilitate the molecular breeding in *Camellia* plants, the database of transcriptome of *C. sinensis* was used for the development of SSR molecular markers. Wu et al. (2012) identified 3767 EST-SSRs potential molecular from *C. sinensis* leaf transcriptome [50]. The transcriptomes of different floral organs (petals, pistils, and stamens) of *C. sinensis* were analyzed and 75,531 uni-genes were assembled and generated 431 novel polymorphic SSR markers [51]. In *Camellia flavida*, 38 polymorphic microsatellite loci were identified based on the transcriptome sequencing and polymorphic alleles between *C. flavida* and *C. nitidissima* were revealed [52]. In *C. oleifera*, molecular markers were also developed and 6949 putative SSR motifs were discovered from a seed transcriptome from which 15 polymorphic genic-SSR markers were verified [53].

## 3. GWAS and QTL Mapping of Key Traits in *Camellia* Plants

Quantitative trait locus mapping is a powerful method for identifying the key genome regions related to the traits of interest [61]. Previously, QTL analyses were performed in many major crops through the marker-assisted breeding methods [62,63,64,65,66]. Genetic linkage maps constructed with various molecular markers are particularly useful for mapping of QTLs. To date, over 10 genetic maps were constructed in tea plants based on different types of markers such as Random Amplification of Polymorphic DNA (RAPD), the Inter-Simple Sequence Repeat (ISSR), Amplified Fragment Length Polymorphism (AFLP), SSR, SNP, and more [51,67,68,69,70,71,72,73,74,75,76,77]. In addition, QTLs for tea plant yield [73], timing of spring bud flush, young shoot color [77], catechins content [75], drought tolerance [67], etc., were mapped. However, these previously mentioned genetic maps were constructed with a low resolution due to the limits in genotyping methods and the size of segregating populations. The research of QTL mapping in tea plants is at a preliminary stage and needs more in-depth developments of molecular markers and linkage maps.

To our knowledge, the genetic map construction and QTL mapping in other *Camellia* species are not reported. There are two potential reasons limiting the study in *Camellia* plants. First, it requires a large population to achieve sufficient segregations of allelic variations [78], which causes vast efforts for data collecting. Second, the commonly appearing polyploidization of *Camellia* cultivars increases the difficulty of fine QTL mapping. In recent years, the high-throughput sequencing technology allows genome-wide genetic variation discovery and genotyping in a highly efficient way [79]. It can greatly increase the resolution of QTL mapping and reduce laborious works [80,81]. Genome-wide association study based on high-throughput sequencing technologies is considered a favorable resolution to explore the allelic variation in a broader scope for extensive phenotypic diversity and as a complementary and powerful tool for connecting the genotype-phenotype map as well. GWAS overcomes the cross-population limitation of QTL mapping and evaluates the association between genotypes and phenotypes of interest based on the natural population with a large number of unrelated individuals. This approach was pioneered in human genetics more than 10 years ago [82] and were now routinely applied in plants including *Arabidopsis* [83] and crops [84,85,86,87,88,89,90,91]. In tree plants, the genome re-sequencing of 544 *Populus trichocarpa* trees and GWAS analysis identified extensive genomic regions related to adaptive trait variation [92]. Due to the abundant genetic diversity and rapid Linkage Disequilibrium (LD) decay, the out-crossing species are suitable for GWAS such as *Camellia* species. However, up to now, GWAS analysis in *Camellia* plants was not reported yet. Due to the complexity of the *Camellia* genome, the genome-wide analysis of *Camellia* population genetics is unfinished and the extensive heterozygosity in the *Camellia* genome makes the polymorphism calling technically challenging. The further efforts of GWAS in cultivated *Camellia* plants are needed. There are some precautions in cultivated *Camellia* GWAS work when the reference genome is available. First, the population size and sequencing coverage are fundamental in an experimental design. Large samples and high coverage will increase GWAS power generally and also enable most allelic variants to be identified. However, it is not always more or better in sampling (especially in plant samples) because the diversity and the individual relationship can greatly affect the effect of GWAS [79]. To achieve cost optimizations, a balance between sequencing depth and sample size should be made. Phenotyping is the most laborious and important work in *Camellia* GWAS. A well-defined trait will increase GWAS power. To ensure the quality of phenotype data, it would be much better to generate phenotype data in several successive years with replications and careful field designs. When a trait is strongly confounded by genetic backgrounds, the power of GWAS will be greatly reduced [79]. An appropriate statistical model can reduce spurious genotype–phenotype associations and increase GWAS power. Computational models including mixed linear, multi-locus mixed, and multi-trait mixed models, which integrate the population structure matrix and pairwise relatedness kinship within populations, have been developed, improved, and optimized to control the rate of spurious genotype–phenotype associations [93,94,95,96,97]. Lastly, in the light of a high-quality reference genome of *Camellia* species, the integration of GWAS and QTL would provide more accurate information of regulatory genes underlying the complex traits. It is expected that, with the support of population, association, and designated omics datasets, the casual loci could be identified. 

## 4. Functional Characterization of Genes Related to Key Pathways of *Camellia* Plants

The enormous amount of transcriptomics studies in *Camellia* plants facilitated the in-depth analysis of genes or gene families related to some fundamental pathways such as floral and fruit development, secondary metabolites, or stress tolerances. For instance, the members of Uridine 5′-diphospho (UDP)-glycosyltransferases in tea (132 transcripts in total) were systematically identified based on transcriptome sequencing [98] and functional analyses revealed that three UDP-glucuronosyltransferases (UGTs) were involved in the biosynthesis of β-glucogallin and glycosylated flavonols [98]. Furthermore, a comparative transcriptomics work that focused on two yellow *Camellia* species (*C. nitidissima* and *C. chuongtsoensis*) suggested that the group C clade of the UGT family was expanded in yellow *Camellia* plants, which was consistent with higher flavonoids levels [32]. These results have highlighted the importance of various genomic datasets in genus *Camellia* to understand the evolutionary adaptations of natural traits.

Yet, current research in *Camellia* plants is hampered by a lack of sufficient molecular tools to validate the functions of underlying genes. Detailed characterization of gene functions in *Camellia* plants remains scarce. To dissect the important economic traits of *Camellia* cultivars, versatile molecular biology toolkits are of significant importance. Currently, transgenic analysis of model plants (e.g., *Arabidopsis*, tobacco) and in vitro protein assays are most commonly used. In a recent study, the enzymatic activities of Lipoxygenases (LOX) from *C. sinensis* were evaluated and the efforts of gene expression and subcellular localization indicated *CsLOX* genes were related to diverse stress responses [99,100]. To study the formation of double flowers in *C. japonica*, homologs of classic ABC model genes were identified and characterized. Through the gene expression and transgenic *Arabidopsis* analyses, it was found that the expression levels of A class genes were positively correlated with the degree of floral doubleness [101] while the C class homolog displayed distinct expression patterns in different types of double flowers in cultivated *Camellia* [102]. It is not clear how the ABC genes are domesticated at the molecular level during double flower formation. The integrative analyses of small RNAs and their targets in doubled *Camellia* cultivars suggested that miRNAs-target regulations played a critical role in the floral organ development of double flowers [33]. Hence, the development of double flowers in *Camellia* required multiple types of regulatory genes including ABC function genes, miRNAs, targets, and other factors.

**Figure 2:**
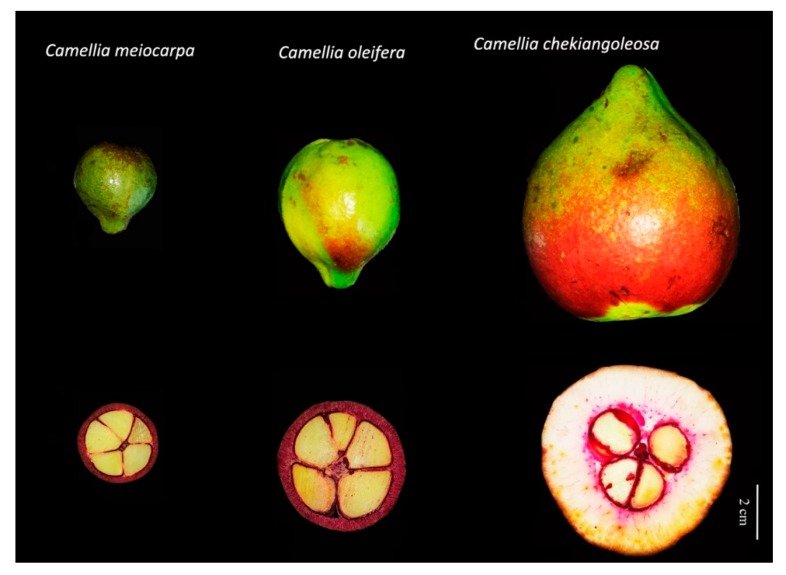
A comparison of oil-*Camellia* fruits and their lignification patterns. The fruits at the stage of rapid enlargement (from left to right, *C. meiocarpa*, *C. oleifera*, and *C. chekiangoleosa*) are presented on the upper panel. The lignification pattern of fruits is revealed by a red color of phloroglucinol-HCl staining on the lower panel.

The *Camellia* fruits were artificially selected mostly for the production of edible oil of seed kernels. It was found that *Camellia* oil is unique in a high level of unsaturated fatty acids and other metabolites beneficial for human health [45]. There was a great diversity of fruit structure of *Camellia* plants used for oil production (Figure 2). 

Besides the plants domesticated from *C. oleifera*, other species with a distinct floral color and flowering time (e.g., *C. chekiangoleosa*, *Camellia polyodonta*, *Camellia reticulata*) were also commonly used for oil production. The comparison of fruit anatomy and a lignification pattern indicated that the genetic alterations of regulatory pathways were underlying the fruit development and ripening during the evolutionary and domestication processes (Figure 2). However, few genes related to *Camellia* fruit development were characterized at present. Taking advantage of the diversity of fruit shape, size, and metabolite in closely related species (Figure 2), which is the fruit development of *Camellia*, served as an ideal system to study the molecular regulation of seed oil biosynthesis, secondary metabolism, lignification, etc.

**Figure 3:**
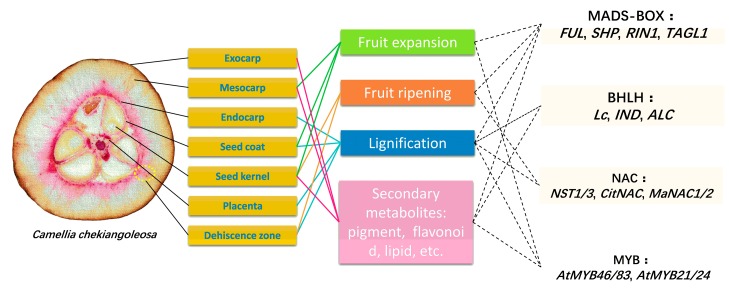
A proposed diagram of transcription factors in the control of fruit development in *C. chekiangoleosa*. A typical fruit of *C. chekiangoleosa* consists of a variety of tissue types that are labeled by yellow boxes. During the development of fruit, some processes including fruit expansion, fruit ripening, lignification, and biosynthesis of secondary metabolites are found to be controlled by several types of transcription factors, according to the studies from diverse plant species. The yellow ellipse indicates the dehiscence zone.

Genetic factors regulating fruit development were uncovered mostly based on studies in model plants such as the *Arabidopsis* silique development and tomato ripening processes [103,104,105] (Figure 3). Currently, several types of transcription factors were found to work coordinately to regulate hormone signaling, lignification, and secondary metabolite biosynthesis during fruit development [106,107,108]. In *Arabidopsis*, three MADS-BOX transcription factors (*FUL*, *SHP1*, and *SHP2*) formed a central regulatory node directing the patterning of fruit development [109]. The homolog of *SHP1/2* in tomatoes was found to regulate a diverse process during fruit development and ripening [110]. The bHLH type transcription factors (*Lc*, *Alcatraz*) were also revealed as key regulators during fruit development in petunia and *Arabidopsis* [111,112,113]. Moreover, NAC (no apical meristem (NAM), activating factor1,2 (ATAF1,2), and cup-shaped cotyledon2 (CUC2)) and MYB transcription factors played important roles in the biosynthesis of the cell wall and secondary metabolites in fruits, which together formed a complex network governing the formation of different fruit morphologies [114,115,116].

The genetic model was found to be useful for investigating the regulation of fruit development in other types of fruits [117,118,119]. The *Camellia* fruit development shares some common processes with other plants such as the lignification of endocarp and flavonoid biosynthesis, which suggests that these transcription factors may also be involved (Figure 3). We examined the structure of *C. chekiangoleosa* fruits and showed that the endocarp and seed coat were lignified at the fruit ripening stage, which were potentially regulated by MADS-BOX and NAC types of transcription factors (Figure 3). The pericarp of *C. chekiangoleosa* was enriched in flavonoids, which could be directed by MYB type transcription factors (Figure 3). It is not clear how the transcriptional regulation participates in the fruit development in *Camellia* plants. Future work of functional analyses of the transcription factors is needed to reveal the diversity of *Camellia* fruit development.

The regulatory genes related to biotic and abiotic stresses in *Camellia* were essential to cultivate new varieties with enhanced environmental resilience and field performance. Presently, regulatory genes, particularly transcription factors and their targets, were discovered extensively in *Camellia* species [16,17,18,120]. However, only a few genes of transcription factors were characterized through transgenic analysis in model species. For example, a basic region/leucine zipper (bZIP) transcription factor in *C. sinensis* (*CsbZIP6*) was studied. Enhanced tolerances of freezing stress were revealed in transgenic *Arabidopsis* plants [121]. Further studies indicated that *CsbZIP6* might induce downstream cold-responsive genes in *Arabidopsis* [121]. A dehydration-responsive element-binding protein (DREB) transcription factor (*CsDREB*) was identified and characterized [122]. Overexpression of *CsDREB* in Arabidopsis revealed that *CsDREB* was involved in salt and drought tolerances via both ABA-dependent and ABA-independent pathways [122]. In *Camellia* plants, the lack of an efficient transformation system hampered the functional analysis of key regulators. However, transient expression or callus transformation approaches might be effective for addressing the regulatory functions of transcription factors related to stress tolerances. 

## 5. Future Perspectives: A Roadmap for *Camellia*

As the demand for *Camellia* products continues to increase, the current breeding work is facing a challenge of obtaining cultivars with enhanced environmental resilience and productivity. To meet the challenges, the large-scale gene expression analysis is becoming a prevalent methodology in the field of *Camellia* research, which greatly facilitates the molecular characterization of important gene families. With the support from large natural or hybridized populations, it is expected that more and more genetic factors or loci will be available for improving the efficiency of breeding new varieties (Figure 1). However, a deep understanding of the molecular mechanism of trait evolution and domestication relies on the capability of functional tools. The facile genetic transformation or transient assay systems in *Camellia* plants are essential to test the hypothesis relevant to candidate gene functions. Lastly, the genome editing technology together with other molecular breeding technologies will enable a precise and targeted innovation of new traits and genetic variations. 

We propose a practical roadmap for *Camellia* research including four key steps. (1) A large-scale collection of germplasms of native and hybrid populations is necessary to advance *Camellia* research. (2) Database initiatives and analyzing platforms for genomics, metabolites, and phenotypes. An international cooperation platform will facilitate the storage, sharing and analysis of high-throughput data, and provide opportunities for generating standardized pipelines for various *Camellia* breeding programs. (3) With the support of reference genomes in *Camellia* plants, the application of genomics tools through whole-genome level analyses (such as QTL mapping, GWAS, and genome re-sequencing) will allow an efficient identification of molecular markers or gene alleles associated with trait variations. (4) Take advantage of the molecular information. The maker assisted selection can help locate important genomic fragments underlying key economic traits. At the same time, the genome selection method is a promising approach to shorten the breeding cycle and integrate elite traits efficiently based on models from whole-genome analyses of molecular makers. Presently, a large number of wild *Camellia* resources are still underutilized and it is expected that, with the support of genomic technologies, the domestication of *Camellia* plants can promote the breeding of elite varieties with enhanced resistances and economic values in the near future.

## Figures and Tables

**Table 1:** A list of recent transcriptomics studies in *Camellia* species related to trait variations and domestication.

Species	Traits	Methods	Key Pathways & Genes	Reference	Database Accessment (from NCBI)
***C. sinensis***	seven tissue types	Transcriptome/Illumina	flavonoid, theanine, and caffeine biosynthesis pathways	[15]	SRX020193, HP701085-HP777243
**Responses of Biotic and Abiotic Stress in *Camellia* Plants**					
***C. sinensis***	Cold acclimation	454 GS-FLX	Cold-related genes	[16]	SRA061043, SRX020193
***C. sinensis***	Same as above	Illumina	AP2/ERF family TFs	[17]	Not found
***C. sinensis***	Leaves with different treatment time of 4 or 38 °C temperature stress	Illumina	WRKY gen family	[18]	Not found
***C. japonica***	mature leaves after 40 d natural low temperature	Illumina	α-linolenic acid and jasmonic acid biosynthesis pathways respond to cold acclimation	[19]	SRP076436
***C. sinensis***	Drought stress and salt stress young leaves	Illumina	Response to drought stress and salt stress	[20]	PRJEB11522
***C. sinensis***	Germination seed of different dehydrate treatment	Illumina	Mechanism of seed dehydration sensitivity	[21]	SRP096975
***C. sinensis***	(NH4)2SO4 treatment buds, leaves and root	Illumina	Nitrogen utilization genes	[22]	SRP077092
***C. sinensis***	Pollen tubes at 25 °C and 4 °C or with NO treatment	Illumina	Potential mechanisms of the participation of NO in pollen tube responses to low temperature	[23]	SRR3270364, SRR3270376, SRR3270829, SRR3270928, SRR3270974, SRR3270993, SRR3270997, SRR3271001, SRR3271002
***C. sinensis***	Leaf tissues of blister blight transition	Illumina	Blister Blight defense	[24]	SRP067826, PRJNA306068
***C. sinensis***	Insect feeding treatment	Illumina	Defense response to insect (*Ectropis. oblique*)	[25]	SRX998353, SRX1543038
**Transcriptomic Analyses in the Control of Secondary Metabolism in *Camellia***					
***C. sinensis***	13 different tissue samples from various organs and developmental stage	Illumina	Secondary metabolite biosynthesis pathways	[26]	SRR1053623, SRR1051214, SRR1054007, SRR1055110, SRR1055182, SRR1054086, SRR1054152, SRR1055108, SRR1055109, SRR1055932, SRR1055933, SRR1055934, SRR1055944
***C. taliensis***	Tender shoots, young leaves, flower buds, and flowers	Illumina	Secondary metabolic biosynthesis pathways	[27]	PRJNA274899
***C. sinensis***	Buds, 2nd leaves, mature leaves and young roots	Illumina	Catechins metabolic pathways	[28]	Not found
***C. sinensis***	Leaf tissues of four tea plant cultivars	Illumina	Catechins biosynthesis pathways	[29]	Not found
***C. asssamica***	Leaf at the purple and green stages	Illumina	Anthocyanin biosynthesis pathway	[30]	Not found
***C. nitidissima***	Floral buds at five different developmental stages	Illumina	Carotenoids and flavonols glucosides biosynthesis pathways	[31]	SRP112181
***C.nitidissima, C. chuongtsoensis***	Young shoot tip or leaves	Illumina	Floral pigmentation and flowering timing	[32]	PRJNA389977, PRJNA400646
**Transcriptomics Studies Related to Floral Patterning, Flowering Timing and Bud Dormancy**					
***C. japonica***	Double flower development	Illumina	ABCE genes, miR156, and targeted squamosa promoter binding protein-likes (SPLs)	[33]	
***C. azalea***	Floral buds	Illumina	Conserved and lineage-specific miRNA	[34]	PRJNA257896, SRP045386
***C. sinensis***	Axillary buds	Illumina	Bud dormancy regulation mechanism	[35]	SRR5040773, SRR5040784
***C. sinensis***	Bud tissues of different developmental stages	ABI PRISM 3730	Dormancy-related genes	[36]	HM003230–HM003378, GW690681–GW691037
***C. azalea***	Three stages of floral bud development:	Illumina	Floral dormancy-associated MADS-box genes	[37]	PRJNA257896, SRP045386
***C. sinensis***	Three opening stages of flowers	Illumina	WRKY, ERF, bHLH, MYB and MADS-box family relate to flower development	[38]	SRR5487532, SRR5487531, SRR5487530, SRR5487529, SRR5487528, SRR5487527,
***C. sinensis***	Two and a buds in July and December	Illumina	Regulatory mechanism of non-deciduous habit in winter	[39]	Not found
***C. sinensis***	Shading leaves (yellow leaf phenotype)	Illumina	Chloroplast development, chlorophyll biosynthesis pathway	[40]	SRX1078570
***C. sinensis***	Adventitious roots from IBA treatment cuttings	Illumina	Potential mechanisms involved in adventitious root formation	[41,42]	PRJNA240661, JK990996-991074
**Transcriptomics in oil *Camellia* Plants**					
***C. oleifera***	Four tissues	454 GS-FLX	Lipid metabolism	[43]	SRR1472854, SRR1472847, SRR1472843, SRR1472842, GBHI00000000
***C. oleifera***	Drought treatment leaves	Illumina	Drought stress genes	[44]	SRP094080
***C. oleifera***	Seed	Illumina	Oil content and fatty acid composition	[45]	SRP111395
***C. oleifera***	Leaves at different elevations of Lu Mountain and Jinggang Mountain	Illumina	Cold acclimation genes	[46]	SRR2146977, SRR2146978, SRR2146979, SRR2146980, SRR2146973, SRR2146974, SRR2146975, SRR2146976
***C. chekiangoleosai***	Seeds, flowers and leaves	454 GS FLX	Anthocyanin biosynthesis pathway genes	[47]	Not found
***C. oleifera, C. meiocarpa***	Mature seed of different moisture content	Illumina	Fatty acid biosynthesis and accumulation pathway	[48]	Not found
***C. oleifera, C. chekiangoleosa, C. brevistyla***	Flower buds	454 GS FLX	Secondary metabolites pathway, *CHS* gene, *FAD2* gene	[49]	HQ704701.1
**Markers Development Based on RNA-sequencing (RNA-seq)**					
***C. sinensis***	Three developmental growth stages leaves	454 GS FLX	Plant growth, development, secondary metabolite, and (expressed sequence tag–simple sequence repeats (EST-SSR) markers	[50]	SRA052793, KA279444–KA304315, HP701085–HP777243
***C. sinensis***	Different flower organizations at the big bud stage	Illumina	SSR Markers, SSR-based linkage map	[51]	SRA053025, GAAC01000001–GAAC01052919
***C. flavida, C. achrysantha***	Flower buds	Illumina	SSR markers	[52]	Not found
***C. oleifera***	Lipid synthesis phase seed	Illumina	SSR markers	[53]	Not found
